# Incremental Value of Genotype Bins over the HAS-BLED Score for the Prediction of Bleeding Risk in Warfarin-Treated Patients with Atrial Fibrillation

**DOI:** 10.1155/2021/9030005

**Published:** 2021-11-23

**Authors:** Jia Liu, Guanyun Wang, Liu'an Qin, Yangxun Wu, Yuting Zou, Xuyun Wang, Ziqian Wang, Yuyan Wang, Shizhao Zhang, Yuxiao Zhang, Tong Yin

**Affiliations:** ^1^Institute of Geriatrics, National Clinical Research Center for Geriatric Diseases, 2^nd^ Medical Center, Medical School of Chinese PLA and Chinese PLA General Hospital, Beijing 10053, China; ^2^Department of Cardiology, 1^st^ Medical Center, Medical School of Chinese PLA, Beijing 10053, China

## Abstract

**Background:**

This study aimed to analyse the role of the HAS-BLED score with the addition of genotype bins for bleeding risk prediction in warfarin-treated patients with atrial fibrillation (AF).

**Methods and Results:**

Consecutive patients with AF on initial warfarin treatment were recruited. For each patient, CYP2C9^*∗*^3 and VKORC1-1639 A/G genotyping was performed to create 3 genotype functional bins. The predictive values of the HAS-BLED score with or without the addition of genotype bins were compared. According to the carrier status of the genotype bins, the numbers of normal, sensitive, and highly sensitive responders among 526 patients were 64 (12.17%), 422 (80.23%), and 40 (7.60%), respectively. A highly sensitive response was independently associated with clinically relevant bleeding (HR: 3.85, 95% CI: 1.88–7.91, *P*=0.001) and major bleeding (HR:3.75, 95% CI: 1.17–11.97, *P*=0.03). With the addition of genotype bins, the performance of the HAS-BLED score for bleeding risk prediction was significantly improved (c-statistic from 0.60 to 0.64 for clinically relevant bleeding and from 0.64 to 0.70 for major bleeding, *P* < 0.01). Using the integrated discriminatory, net reclassification improvement, and decision curve analysis, the HAS-BLED score plus genotype bins could perform better in predicting any clinically relevant bleeding than the HAS-BLED score alone.

**Conclusions:**

Genotypes have an incremental predictive value when combined with the HAS-BLED score for the prediction of clinically relevant bleeding in warfarin-treated patients with AF.

## 1. Introduction

Patients with atrial fibrillation (AF) are at a high risk for stroke and systemic embolism, which could be substantially reduced with the administration of oral anticoagulants, including vitamin K antagonists and nonvitamin K antagonist oral anticoagulants (NOACs) [1–3]. As a vitamin K antagonist, warfarin has been widely used in the prevention and treatment of thromboembolic diseases. However, due to the risks of serious and fatal bleeding complications, the use of warfarin should therefore be based on a careful assessment of both thromboembolic and bleeding risks [4]. In comparison with warfarin, NOACs could reduce the risk of thromboembolic events, with lower mortality and half the risk of intracranial haemorrhage [3, 5–7]. The benefit of NOACs was confirmed to be greater in patients with poor control of warfarin than in those with good control [8]. Therefore, early prediction of the poor control of warfarin anticoagulation is vital for personalized selection between warfarin and NOACs in AF patients who need anticoagulation treatment [9]. The HAS-BLED bleeding risk score (hypertension (systolic blood pressure >160 mmHg), abnormal liver/renal function (creatinine ≥200 *μ*mol/L), stroke, bleeding history or predisposition, labile international normalized ratio (INR) (INR in range <60% of the time), elderly (>65), and concomitant drugs/alcohol) [10, 11] is the most widely validated model in AF and non-AF populations [12]. It has been shown to outperform CHADS_2_ (cardiac failure, hypertension, age, diabetes, and stroke (double)) or CHA_2_DS_2_-VASc in predicting bleeding risk [13]. Further validation studies have confirmed that the HAS-BLED score is an effective predictor of bleeding in warfarin-naive patients and in those administered warfarin plus aspirin or nonwarfarin treatment [14, 15]. The estimation of bleeding risk in AF patients undergoing anticoagulation therapy has been recommended by current guidelines [3, 16–18]. However, the predictive capacity of the HAS-BLED score for bleeding risk in warfarin-anticoagulated patients remains modest even with the incorporation of the labile INR [14, 19].

In recent decades, strong evidence has shown that the genotypes of two genes encoding the major enzyme for the warfarin metabolism (cytochrome P4502C9 and CYP2C9) and the target protein of warfarin (vitamin K epoxide reductase, VKORC1) are clearly associated with the variability of warfarin dose and sensitivity (indicating increased risk of bleeding under warfarin treatment) [20–22]. Specifically, the mechanisms behind the association showed that the CYP2C9^*∗*^2 and ^*∗*^3 genotypes result in reduced metabolism of the highly active warfarin [23], and the VKORC1–1639G to A variant could alter a transcription factor binding site, which leads to reduced levels of the molecular target of warfarin [24].

Genotype-guided warfarin dosing has been confirmed to improve warfarin anticoagulation [25–27], including in Asian adults [28]. In addition, findings from the ENGAGE AF-TIMI 48 trial showed that patients carrying warfarin-sensitive (carriers of CYP2C9^*∗*^1^*∗*^1 + VKORC1-1639A/A or CYP2C9^*∗*^1^*∗*^3 + VKORC1-1639G/A) or highly sensitive (carriers of CYP2C9^*∗*^1^*∗*^3 + VKORC1-1639A/A, CYP2C9^*∗*^3^*∗*^3 + VKORC1-1639A/A, CYP2C9^*∗*^3^*∗*^3 + VKORC1-1639G/A, or CYP2C9^*∗*^3^*∗*^3 + VKORC1-1639G/G) genotype functional bins of CYP2C9 and VKORC1 spend more time over-anticoagulated and have higher rates of bleeding events than noncarriers during warfarin treatment, especially in the early period after the start of treatment [29]. The trial also indicated that the CYP2C9 and VKORC1 genotypes could provide a significant benefit for the identification of patients who are predisposed to bleeding with warfarin.

Previously, a novel biomarker-based ABC bleeding risk score has been generated and validated with superior bleeding risk prediction capability compared to the HAS-BLED score which includes only clinical variables [30]. Genotype is a kind of biomarker; therefore, we hypothesized that with the addition of the genotype bins related to the response to warfarin, the predictive efficacy of the HAS-BLED score might be improved. The present study aimed to analyse the role of warfarin sensitivity-related genotype bins in addition to the HAS-BLED score for the prediction of major bleeding events in warfarin-treated patients.

## 2. Methods

### 2.1. Patient Recruitment

Consecutive patients with AF on initial warfarin treatment were recruited from the Chinese PLA General Hospital, Beijing, China, between January 2015 and June 2016. The Ethical Review Committee of the Chinese PLA General Hospital approved this prospective observational study. Each patient provided written informed consent in accordance with the Declaration of Helsinki. The included consecutive patients with AF were over 18 years old, prescribed warfarin for ≥3 months, and willing to undergo genetic testing. Patients were excluded from the study if they were administrated with low-intensity warfarin anticoagulation. As the majority of patients were Han Chinese, patients of other ethnicities were not included in the study.

### 2.2. Warfarin Anticoagulation and Follow-Up

Patients who received standard warfarin anticoagulation were controlled with a target INR of 2.0–3.0. Since bleeding complications and switching to NOACs are more likely to occur within the initial stage of warfarin anticoagulation, a follow-up interval of 3 months was required for the study. Experienced physicians who were unaware of the genotypes during the whole treatment period determined the frequency of INR measurements and warfarin dosing. The time in therapeutic range (TTR) based on the measured INRs in 3 months was calculated using the Rosendaal method [31]. An average TTR of <60% was considered a labile INR or poor control of warfarin anticoagulation [14]. Patient demographic and clinical characteristic data associated with warfarin treatment were collected from electronic medical records, patient self-reports, and a review by a trained nurse. Stroke risk at baseline was assessed for each patient using the CHA_2_DS_2_-VASc score [32].

The principal safety outcome of the study was any clinically relevant bleeding event, including major bleeding and clinically relevant nonmajor bleeding, defined according to the derivation and validation studies of the HAS-BLED score. Major bleeding events were defined as any bleeding requiring hospitalization or causing a decrease in the haemoglobin level of more than 2 g/L or requiring blood transfusion. Clinically relevant nonmajor bleeding was defined as that which did not meet the major bleeding criteria but that satisfied defined criteria (including haematuria, haematemesis, repetitive epistaxis for more than 5 minutes at least twice in 24 hours, or subcutaneous haematomas of more than 25 cm^2^ if spontaneous or more than 100 cm^2^ if after trauma) [10, 14, 33]. All suspected bleeding events were classified by a central adjudication committee unaware of the treatment assignments.

### 2.3. Genotyping Analysis

Genotyping was undertaken using the Sanger sequencing method as described previously for the detection of warfarin sensitivity-related genotypes in Chinese individuals, including CYP2C9^*∗*^3 (rs1057910) and VKORC1-1639G/A (rs9923231) [34]. The patients were grouped according to the genotype functional bins that corresponded to the FDA categories for response on the updated warfarin label: normal responders (carriers of CYP2C9^*∗*^1^*∗*^1 + VKORC1-1639G/G, CYP2C9^*∗*^1^*∗*^1 + VKORC1-1639G/A, or CYP2C9^*∗*^1^*∗*^3 + VKORC1-1639G/G), sensitive responders (carriers of CYP2C9^*∗*^1^*∗*^1 + VKORC1-1639A/A or CYP2C9^*∗*^1^*∗*^3 + VKORC1-1639G/A), and highly sensitive responders (carriers of CYP2C9^*∗*^1^*∗*^3 + VKORC1-1639A/A, CYP2C9^*∗*^3^*∗*^3 + VKORC1-1639 A/A, CYP2C9^*∗*^3^*∗*^3 + VKORC1-1639G/A, or CYP2C9^*∗*^3^*∗*^3 + VKORC1-1639G/G) [29].

### 2.4. HAS-BLED Score Calculation

The HAS-BLED score was calculated with the addition of 1 point for each variable, including hypertension, abnormal renal function, abnormal liver function, stroke, bleeding history or predisposition, labile INR, elderly age, and concomitant drugs/alcohol. According to the current guidelines [3], a HAS-BLED score of ≥3 indicates that the patient is at high risk of bleeding and requires caution and regular review.

### 2.5. Statistical Analysis

Continuous variables, presented as the mean ± SD, were tested for normality using the Kolmogorov–Smirnov test. Categorical variables are presented as percentages. The baseline characteristics of patients receiving warfarin were compared among normal, sensitive, and highly sensitive responders. We used the chi-square test to compare the bleeding outcomes between normal responders and sensitive and highly sensitive responders. Multivariate logistic regression analysis was used to analyse the independent role of responders to warfarin according to genotype bins along with the HAS-BLED score in regard to major bleeding complications. We constructed a receiver operating characteristic (ROC) curve to evaluate the predictive performance of responders to warfarin according to genotype bins in addition to the HAS-BLED score for bleeding events. The comparison between the performance of the HAS-BLED score with or without the addition of genotypes was achieved by the calculation of the c-statistic, integrated discriminatory improvement (IDI), net reclassification improvement (NRI), and decision curve analysis (DCA). The c-statistic was calculated by ROC analysis. IDI and NRI were performed using the “PredictABEL” packages. DCA was performed using the “rmda”and “ggDCA” packages. All calculations were performed with SPSS version 22.0 (SPSS Inc, Chicago, Illinois), MedCalc v. 18.2.1 (MedCalc Software bvba, Ostend, Belgium), and *R* Software v.3.2.5 (R Foundation for Statistical Computing, Vienna, Austria).

## 3. Results

### 3.1. Baseline Demographic and Clinical Characteristics

A total of 584 eligible patients with AF who were treated with warfarin were enrolled in the study. Of these, 17 patients were excluded due to the alternative administration of NOACs within 3 months, 38 were excluded due to loss of follow-up, and 3 were excluded due to death within 3 months. Finally, a total of 526 patients with AF who were on warfarin were recruited; among them, 290 (55.13%) were male, and the mean age was 60.63 ± 11.05 years. The average stable therapeutic dose of warfarin was 2.89 ± 1.06 (range: 0.625–8 mg/day), and 143 (27.18%) patients had a TTR of <60% within the 3-month follow-up. The average CHA_2_DS_2_-VASc score was 2.22 ± 1.53 (range: 0–8), and the average HAS-BLED score was 1.57 ± 1.20 (range: 0–7). The baseline demographic and clinical characteristics of the included patients are given in Table 1. TTR is related to the quality of warfarin anticoagulation and is an important index; the average TTR values are given in Table 1.

### 3.2. Contribution of Genotype Bins and HAS-BLED Score to Bleeding Events

**Figure 1:**
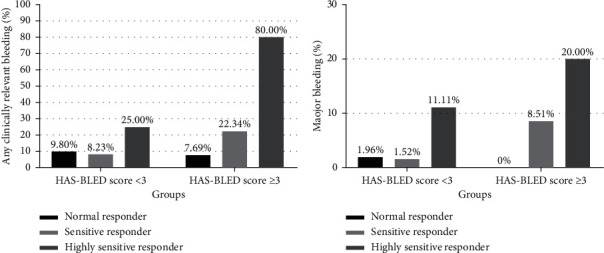
Bleeding events according to the genotype bins defining warfarin response and the HAS-BLED score. Highly sensitive responders were defined as carriers of the genotype bins CYP2C9 ^*∗*^1^*∗*^3 + VKORC1-1639A/A, CYP2C9^*∗*^3^*∗*^3 + VKORC1-1639 A/A, CYP2C9^*∗*^3^*∗*^3 + VKORC1-1639G/A, or CYP2C9^*∗*^3^*∗*^3 + VKORC1-1639G/G.

All patients were successfully genotyped, with 64 (12.17%) categorized as normal responders, 422 (80.23%) as sensitive responders, and 40 (7.60%) as highly sensitive responders to warfarin according to the genotype bins of CY2C9^*∗*^3 and VKORC1-1639A *>* G. During the 3-month follow-up, any clinically relevant bleeding event occurred in 67 (12.73%) patients, with major bleeding events occurring in 18 (3.42%) patients. Highly sensitive responders experienced significantly higher rates of both clinically relevant bleeding (32.5%) and major bleeding (10%) than sensitive (11.35% for clinically relevant and 3.08% for major) and normal responders (9.37% for clinically relevant and 1.56% for major) (Table 2, Figure 1). Compared with patients who had a HAS-BLED score of 0–2, patients with a HAS-BLED score of ≥3 had a significantly increased risk of major bleeding and any clinically relevant bleeding (Table 2; Figure 1). After multiple logistic regression analysis, both highly sensitive responders and the HAS-BLED score were independent risk factors for major bleeding (HR: 3.75, 95% CI: 1.17–11.97, *P*=0.001; HR:3.93, 95% CI: 1.52–10.16, *P*=0.005) and any clinically relevant bleeding (HR: 3.85, 95% CI: 1.88–7.91, *P*=0.001; HR:2.75, 95% CI: 1.60–4.74, *P*=0.001) (Table 3).

### 3.3. Performance of the HAS-BLED Score Plus Genotype Bins for Bleeding Risk Prediction

**Figure 2:**
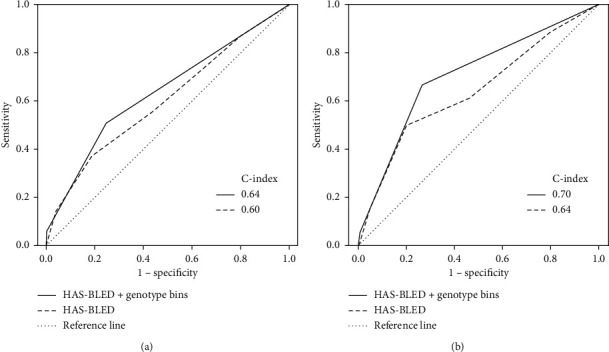
Receiver operating characteristic curves of the HAS-BLED score with genotype bins defining highly sensitive responders for the prediction of bleeding events. HAS-BLED score plus genotype bins indicates the addition of the genotype bins related to highly sensitive responders (CYP2C9 ^*∗*^1^*∗*^3 + VKORC1-1639A/A, CYP2C9^*∗*^3^*∗*^3 + VKORC1-1639 A/A, CYP2C9^*∗*^3^*∗*^3 + VKORC1-1639G/A, or CYP2C9^*∗*^3^*∗*^3 + VKORC1-1639G/G) to HAS-BLED score ≥3. (a) Any clinically relevant bleeding. (b) Major bleeding.

**Figure 3:**
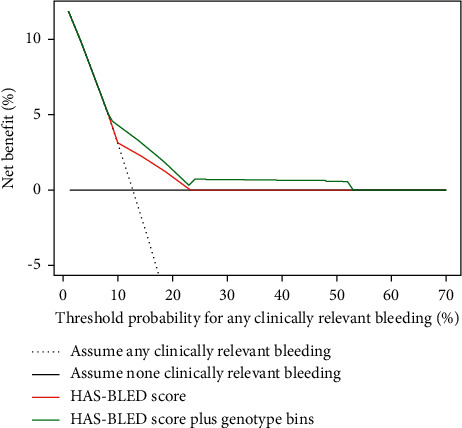
Decision curve analyses (DCAs) of the HAS-BLED score alone and the HAS-BLED score combined with genotype bins. This analysis shows the clinical usefulness of theHAS-BLED score alone and the HAS-BLED score combined with genotype bins based on a continuum of potential thresholds for all clinically relevant bleeding events (*x*-axis) and the net benefit of using the model to stratify AF patients at risk (*y*-axis) relative to assuming that none of the patients will have a clinically relevant bleeding event. This DCA evaluated the clinical usefulness of the HAS-BLED score combined with genotype bins for all clinically relevant bleeding events in real practice. The use of the HAS-BLED score combined with genotype bins implied a better net benefit than the HAS-BLED score alone.

In patients with both a highly sensitive response and a HAS-BLED score ≥3, the risks increased for both clinically relevant bleeding (HR:3.39, 95% CI: 2.01–5.73, *P*=0.001) and major bleeding (HR: 5.53, 95% CI: 2.03–15.01, *P*=0.001) (Table 3). With the addition of a highly sensitive response to the HAS-BLED score, the ROC curves showed an improved ability for the discrimination of all clinically relevant bleeding (c-statistic from 0.60 to 0.64, *P*=0.01), especially the major bleeding risk (c-statistic: from 0.64 to 0.70, *P* < 0.001) (Figure 2). Based on the analyses of IDI and NRI, the HAS-BLED score plus genotype bins showed an increasing sensitivity (3.35%, *P*=0.001) and a significant positive reclassification (55.68%; *P*=0.003) for any clinically relevant bleeding (Table 4). Using DCA, the addition of genotype bins to the HAS-BLED score showed a better clinical benefit than that from the HAS-BLED score alone (Figure 3).

## 4. Discussion

In the present study, we found that the genotype functional bins of CYP2C9^*∗*^3 and VKORC1-1639 A > G provided on the FDA warfarin label [35] affect bleeding outcomes in warfarin-treated patients with AF. The model with the combination of a genotype bin (highly sensitive responder) and the HAS-BLED score yielded a significantly better prognostic performance than the HAS-BLED score alone. The results of the current study are consistent with those of a previous prespecified pharmacogenetic study based on the randomized, double-blind trial of ENGAGE AF-TIMI 48 [29]. This result suggested that clinical and genetic data-based bleeding risk stratification has the potential for personalized anticoagulation in warfarin-treated patients with AF.

The HAS-BLED score is a relatively simple tool integrating clinical predictors to estimate the bleeding risk in patients with AF receiving anticoagulation therapy [10]. However, the predictive performance is moderate in warfarin-treated patients [14, 19, 36, 37], which indicates that additional risk factors could contribute to bleeding complications. We found that the genotype bin-defined highly sensitive response played an independent role in the risk of clinically relevant bleeding. Patients with both a HAS-BLED score ≥3 and a genetic status of highly sensitive responder would have an approximately 6-fold increased risk of major bleeding when treated with warfarin. As suggested, NOACs would be a better treatment for these patients, since compared with warfarin, NOACs were associated with a greater reduction in bleeding risk [29]. Therefore, it is reasonable to combine the HAS-BLED score with a highly sensitive response for the prediction of bleeding risk in warfarin-treated patients. Regarding the clinical implications, the new model could facilitate decision-making by discriminating AF patients with a high risk for clinically relevant bleeding (HAS-BLED score ≥3 and highly sensitive responders) on warfarin treatment who would be better administered NOACs to achieve acceptable anticoagulation control. In view of the broad adoption of NOACs in clinical practice, warfarin continues to play a role in patients with contraindications or lack of access to NOACs. Moreover, warfarin is currently the main treatment with established safety in AF patients with rheumatic mitral valve disease and/or an artificial heart valve [3]. In such circumstances, warfarin-sensitive genotype bins are valuable for the selection of patients who might be suitable for warfarin treatment or who might derive a greater safety benefit from NOACs than warfarin.

Several limitations should be mentioned in regard to the present study. First, the present study was a single-centre observational study, which may not be fully representative of all clinical practice. Further external validation in broader and ethnically diverse patients is needed to assure the generalization of the results. Second, the present study was not powerful enough to observe an improvement of the new model compared to the HAS-BLED score, with the DCA showing only a relatively small improvement of clinical benefit. Therefore, to confirm the clinical value of genotype bins, further large-scale studies should be carried out to explore the contribution and cost-effectiveness of warfarin-sensitive genotype bins plus the HAS-BLED score. Third, the hypothesis that with the addition of genotypes, the HAS-BLED score would be implicated in the personalized selection of VKA or NOACs in AF patients remains unproven until it can be validated prospectively in a well-designed randomized clinical trial.

In conclusion, the warfarin highly sensitive responder genotype bin could moderately improve the performance of the HAS-BLED score for the prediction of bleeding complications in warfarin-treated Chinese patients with AF.

## Figures and Tables

**Table 1:** Baseline demographic and clinical characteristics.

Baseline characteristics	Normal responder (*n* = 64)	Sensitive responder (*n* = 422)	Highly sensitive responder (*n* = 40)	*P*
Male sex, *n* (%)	37 (57.81%)	230 (54.50%)	22 (55%)	0.88
Age, mean ± SD	58.91 ± 9.94	60.94 ± 11.08	60.52 ± 12.33	0.41

Comorbidities				
Hypertension, *n* (%)	27 (42.18%)	168 (39.81%)	14 (35%)	0.76
Hypercholesterolemia, *n* (%)	13 (20.31%)	55 (13.03%)	6 (15%)	0.30
Diabetes, *n* (%)	10 (15.62%)	48 (11.37%)	7 (17.5%)	0.38
Previous stroke, *n* (%)	12 (18.75%)	60 (14.22%)	3 (7.5%)	0.28
Coronary artery disease, *n* (%)	23 (35.94%)	100 (23.70%)	11 (27.5%)	0.11
Peripheral arterial disease, *n* (%)	7 (10.94%)	64 (15.17%)	3 (7.5%)	0.31
Hepatic impairment, *n* (%)	2 (3.12%)	22 (5.21%)	2 (5%)	0.61
Renal impairment, *n* (%)	10 (15.62%)	32 (7.58%)	5 (12.5%)	0.08
Valvular heart disease, *n* (%)	28 (43.75%)	180 (42.65%)	17 (42.5%)	0.99
Tobacco, *n* (%)	8 (12.5%)	76 (18.01%)	7 (17.5%)	0.58
Alcoholic, *n* (%)	8 (12.5%)	63 (14.93%)	6 (15%)	0.88

Medications during follow-up				
Amiodarone, *n* (%)	15 (23.44%)	87 (20.62%)	8 (20%)	0.87
Antiplatelets, *n* (%)	7 (10.94%)	25 (5.92%)	2 (5%)	0.30
Digoxin, *n* (%)	20 (31.25%)	123 (29.15%)	9 (22.5%)	0.61
Beta-blockers, *n* (%)	21 (32.81%)	112 (26.54%)	9 (22.5%)	0.46
Calcium channel blockers, *n* (%)	18 (28.12%)	93 (22.04%)	10 (25%)	0.53
CHA_2_DS_2_-VASc score, mean ± SD	2.28 ± 1.74	2.23 ± 1.52	1.93 ± 1.27	0.45
HAS-BLED score, mean ± SD	1.70 ± 1.23	1.57 ± 1.19	1.38 ± 1.25	0.40
Average TTR	64.04 ± 19.42	69.88 ± 18.18	66.92 ± 18.89	0.22

^*∗*^SD, standard deviation; TTR, time in therapeutic range.

**Table 2:** Bleeding events according to the HAS-BLED score and genotype bins defining warfarin response.

	All patients (*n* = 526)	Any clinical relevant bleeding event (*n* = 67)		Major bleeding event (*n* = 18)	
		*n* (%)	*P*	*n* (%)	*P*
HAS-BLED score					
0	102 (19.39)	9 (8.82)		2 (1.96)	
1	177 (33.65)	18 (10.17)		4 (2.26)	
2	135 (25.67)	14 (10.37)		3 (2.22)	
3	82 (15.59)	16 (19.51)		6 (7.32)	
≥4	30 (5.70)	10 (33.33)	^*∗*^0.001	3 (10)	^*∗*^0.06
HAS-BLED score <3	414 (78.71)	41 (9.90)		9 (2.17)	
HAS-BLED score ≥3	112 (21.29)	26 (23.21)	^#^0.0003	9 (8.04)	^#^0.005

Genotype bins defining warfarin response					
Normal responder	64 (12.17)	6 (9.37)		1 (1.56)	
Sensitive responder	422 (80.23)	48 (11.37)		13 (3.08)	
Highly sensitive responder	40 (7.60)	13 (32.5)	^§^0.0002	4 (10.00)	^§^0.03

^*∗*^ *P* value indicating the comparison of the bleeding incidence rate among the different accumulated points of the HAS-BLED score. ^#^Comparison between patients with a HAS-BLED score <3 and patients with a HAS-BLED score ≥3. ^§^Comparison among patients with different genotype bins defining warfarin response (normal responders (carriers of CYP2C9^*∗*^1^*∗*^1 + VKORC1-1639G/G, CYP2C9^*∗*^1^*∗*^1 + VKORC1-1639G/A, or CYP2C9^*∗*^1^*∗*^3 + VKORC1-1639G/G), sensitive responders (carriers of CYP2C9 ^*∗*^1^*∗*^1 + VKORC1-1639A/A or CYP2C9 ^*∗*^1^*∗*^3 + VKORC1-1639G/A), and highly sensitive responders (carriers of CYP2C9 ^*∗*^1^*∗*^3 + VKORC1-1639A/A, CYP2C9^*∗*^3^*∗*^3 + VKORC1-1639 A/A, CYP2C9^*∗*^3^*∗*^3 + VKORC1-1639G/A, or CYP2C9^*∗*^3^*∗*^3 + VKORC1-1639G/G).

**Table 3:** Independent contribution of the HAS-BLED score and genotype bins defining warfarin response to bleeding events.

Variable	Any clinically relevant bleeding		Major bleeding	
	Hazard ratio (95% CI)	^a^Adjusted *P*	Hazard ratio (95% CI)	^a^Adjusted *P*
HAS-BLED score ≥3	2.75 (1.60–4.74)	0.001	3.93 (1.52–10.16)	0.005
Highly sensitive responder	3.85 (1.88–7.91)	0.001	3.75 (1.17–11.97)	0.03
HAS-BLED score ≥3 plus highly sensitive responder	3.39 (2.01–5.73)	0.001	5.53 (2.03–15.01)	0.001

Highly sensitive responders were defined as carriers of the genotype bins CYP2C9 ^*∗*^1^*∗*^3 + VKORC1-1639A/A, CYP2C9^*∗*^3^*∗*^3 + VKORC1-1639 A/A, CYP2C9^*∗*^3^*∗*^3 + VKORC1-1639G/A, or CYP2C9 ^*∗*^3^*∗*^3 + VKORC1-1639G/G. ^a^Adjusted for age, sex, alcohol intake, aimed INRs, concomitant medications, and comorbidity conditions.

**Table 4:** C-indices of the ROC curves, ROC curve comparison, IDI and NRI of the HAS-BLED score plus genotype bins defining warfarin response compared to the HAS-BLED score.

	C-index	95% CI	*P*	*P* ^*∗*^	IDI (%)	*P*	NRI (%)	*P*
Any clinically relevant bleeding								
HAS-BLED score plus genotype bins	0.64	0.60–0.69	**<0.001**	**0.03**	3.35	**0.001**	55.68	**<0.001**
HAS-BLED score	0.60	0.56–0.64	**0.001**					

Major bleeding								
HAS-BLED score plus genotype bins	0.70	0.66–0.74	**<0.001**	0.21	1.43	**0.210**	30.30	0.130
HAS-BLED score	0.64	0.61–0.69	**0.020**					

HAS-BLED score plus genotype bins indicate the addition of the genotype bins related to highly sensitive responder (CYP2C9^*∗*^1^*∗*^3 + VKORC1-1639A/A, CYP2C9^*∗*^3^*∗*^3 + VKORC1-1639A/A, CYP2C9^*∗*^3^*∗*^3 + VKORC1-1639G/A, or CYP2C9^*∗*^3^*∗*^3 + VKORC1-1639G/G) to HAS-BLED score ≥3. CI, confidence interval; IDI, integrated discriminatory improvement; NRI, net reclassification improvement. ^*∗*^For c-index comparison.

## Data Availability

The data used to support the findings of this study are available from the corresponding author upon request.
